# Rasch analysis of the hospital anxiety and depression scale among Chinese cataract patients

**DOI:** 10.1371/journal.pone.0185287

**Published:** 2017-09-26

**Authors:** Xianchai Lin, Ziyan Chen, Ling Jin, Wuyou Gao, Bo Qu, Yajing Zuo, Rongjiao Liu, Minbin Yu

**Affiliations:** State Key Laboratory of Ophthalmology, Zhongshan Ophthalmic Center, Sun Yat-sen University, Guangzhou, China; Universita Cattolica del Sacro Cuore Sede di Roma, ITALY

## Abstract

**Purpose:**

To analyze the validity of the Hospital Anxiety and Depression Scale (HADS) among Chinese cataract population.

**Methods:**

A total of 275 participants with unilateral or bilateral cataract were recruited to complete the Chinese version of HADS. The patients' demographic and ophthalmic characteristics were documented. Rasch analysis was conducted to examine the model fit statistics, the thresholds ordering of the polytomous items, targeting, person separation index and reliability, local dependency, unidimentionality, differential item functioning (DIF) and construct validity of the HADS individual and summary measures.

**Results:**

Rasch analysis was performed on anxiety and depression subscales as well as HADS-Total score respectively. The items of original HADS-Anxiety, HADS-Depression and HADS-Total demonstrated evidence of misfit of the Rasch model. Removing items A7 for anxiety subscale and rescoring items D14 for depression subscale significantly improved Rasch model fit. A 12-item higher order total scale with further removal of D12 was found to fit the Rasch model. The modified items had ordered response thresholds. No uniform DIF was detected, whereas notable non-uniform DIF in high-ability group was found. The revised cut-off points were given for the modified anxiety and depression subscales.

**Conclusion:**

The modified version of HADS with HADS-A and HADS-D as subscale and HADS-T as a higher-order measure is a reliable and valid instrument that may be useful for assessing anxiety and depression states in Chinese cataract population.

## Introduction

Cataract is the most common cause of visual impairment in China[1–3]. Visual loss and visual disability significantly impact mental health and affect the quality of life in the aging population[4–6]. Depression and anxiety are among the major mental health problems in the elderly, especially in visually impaired older adults[5]. It is estimated that 10% elderly community-dwelling residents and 15% to 25% of hospitalized patients in China experience major depression disorder[7]. The prevalence of subthreshold depression (32.2%) and subthreshold anxiety (15.6%) among patients is twice as high as the prevalence in general elderly populations[5].

Vision impairment due to cataract has been significantly associated with depression and anxiety in older adults[8–10]. In a community-based survey of 4611 Chinese adults aged over 60 years using the 9-item Patient Health Questionnaire (PHQ-9) depression scale, adults with cataract had higher odds of having depressive symptoms compared with those without cataract[8]. A study of 662 individuals aged over 70 years in Australia using the Goldberg scales (GADS) found anxiety and depression symptoms were associated with cataract[9]. Palagyi et al demonstrated a high prevalence of depressive symptoms in older persons with cataract[10]. Several studies have examined the impact of cataract surgery on depression and anxiety[11–13]. However, few studies have evaluated the association of anxiety and depression with cataract in Chinese population.

The Hospital Anxiety and Depression Scale (HADS) is an useful instrument for screening anxiety and depression[14]. The Chinese version of the HADS has been developed and validated previously [15, 16]. So far, no study used the HADS in Chinese cataract patients. The question has been raised as to the suitability of the HADS measures in Chinese cataract population. The Rasch model is a psychometric method that ensures assessments of reliability and validity of the scaling properties of an instrument[17–19]. Rasch validation of the HADS has been proven useful in dry eye patients[20]. In the current study, Rasch analysis was used to validate the Chinese version of the HADS in cataract patients.

## Methods

### Study population

A sample of 275 participants with unilateral or bilateral cataract over the age of 40 years was recruited from the Zhongshan Ophthalmic Center, Sun Yat-sen University, Guangzhou, Southern China between April 2016 and April 2017. Participants with first eye operated previously were excluded. All patients completed the Hospital Anxiety and Depression Scale (HADS) questionnaire and an additional questionnaire for information about patients' ophthalmic and demographic characteristics. Clinical information was collected by the examining ophthalmologists. Written informed consent was obtained from all participants. The study adhered to the Declaration of Helsinki and was conducted after obtaining ethical approval from the Zhongshan Ophthalmic Center Institutional Review Board.

### HADS questionnaire

The HADS is a self-administered scale with 7 anxiety and 7 depression items rated on a scale from 0 to 3[14]. The Chinese version of the HADS was used in the present study[15, 16].

### Rasch analysis

The Rasch measurement model was used to construct validity of the HADS[21]. The Rasch model estimates a person’s ability in relation to item difficulty expressed in log odds units (logits) on a single continuum scale. For this analysis, participants with higher ability and items of greater difficulty were located on the negative side of the continuum scale and vice versa[22].

For Rasch analysis, a minimum sample size of 243 will provide useful and stable estimations of items and person locations irrespective of scale targeting[23]. Rasch analysis was performed on anxiety and depression subscales as well as HADS-Total score respectively. The Winsteps program (Version 3.92.1, Winsteps, Beaverton, Oregon, USA) was used for Rasch analysis using the Andrich rating scale model for HADS-Anxiety and partial credit model for HADS-Depression and HADS-Total score.

#### Category threshold order

The category probability curves were used to assess the threshold ordering of polytomous items. The extent to which responses to the items are consistent with the metric estimate of the underlying construct is indicated by an ordered set of response thresholds for each of the items. When disordered thresholds occur or two response categories on an item are difficult to be discriminated, collapsing the categories into one response option can improve scale fit to the Rasch model[17, 24].

#### Rasch model fit

The item fit statistics are expressed in infit and outfit mean square (MNSQ) statistics which is based on the chi-square statistic with each observation weighted by its statistical information (model variance). A range of 0.7 to 1.3 is used as a criterion of good fit[24, 25].

The Likelihood ratio test was used to compare revised model with original model. Winsteps reports global fit statistics and approximates global log-likelihood chi-squared statistic. Deviance statistics for comparing different models are the difference between the chi-squares of two analyses, with d.f. of the difference between the number of free parameters being estimated.

Free parameters = non-extreme items + non-extreme persons—1 + (categories in estimated rating-scale structures—2 * rating-scale structures) [26].

#### Targeting

Targeting refers to how well the difficulty of items matches the abilities of the study sample. The standard error of the person measure was used for the assessment. The cut-off points were defined: fair targeting as 1–2 error, good targeting as <1 error and very good targeting as <0.5 error [27].

#### Differential item functioning (DIF)

The analysis of DIF including uniform and non-uniform was performed to identify significant differences of the response on an item by subgroups of the demographic characteristics. We assessed DIF by Age (≤70; > 70), Gender and Education (primary school and lower; junior school and higher). DIF differences were presented. Notable DIF was defined as the difference >1.0 logits[28].

#### Local dependency

Local dependency was identified with paired standardized residual correlations between items exceeding 0.30. If the problem occurs, the dependent items are recommended to be added together into one item[29].

#### Measurement precision assessed by person separation reliability (PSR) and separation index (PSI)

Person separation is used to classify people. Low person separation with a relevant person sample implies that the instrument may not be sensitive enough to distinguish between high and low performers. More items may be needed. Reliability means reproducibility of relative measure location. A PSR of ≥0.80 (PSI≥2.00) indicates that the instrument can distinguish the study population into 2–3 levels disability[30].

#### Unidimensionality

Unidimensionality of the Rasch model is assessed by independent t-tests for each person. The percentage of such tests outside the ± 1.96 range should be less than 5% which is required to indicate a unidimensional scale[24, 31].

In principal component analysis of the residuals (PCA), 60% of the variance explained by the raw data is considered unidimensionality[25, 28]. An eigenvalue in the first contrast in the residuals > 2.0 as well as indicates that a second construct is needed to be measured[26].

## Results

### The demographic characteristics of the study population

A total of 275 people participated in the study (Table 1). The mean age (SD) of participantswas70.5 (11.1). 38.9% were male and 48.5% of participants had low level of education.

**Table 1:** Characteristics of the participants.

Characteristics	n (%), Mean (SD)	Missing, n (%)
**Total**	275	
**Age (year), n (%)**		2 (0.73)
≤70	127 (46.5)	
>70	146 (53.5)	
Mean (SD)	70.5 (11.1)	
**Male sex, n (%)**	107 (38.9)	0 (0.00)
**Education, n (%)**		9 (3.27)
Primary school and lower	129 (48.5)	
Junior school and higher	137 (51.5)	

### Rasch analysis of HADS-Anxiety

**Fig 1:**
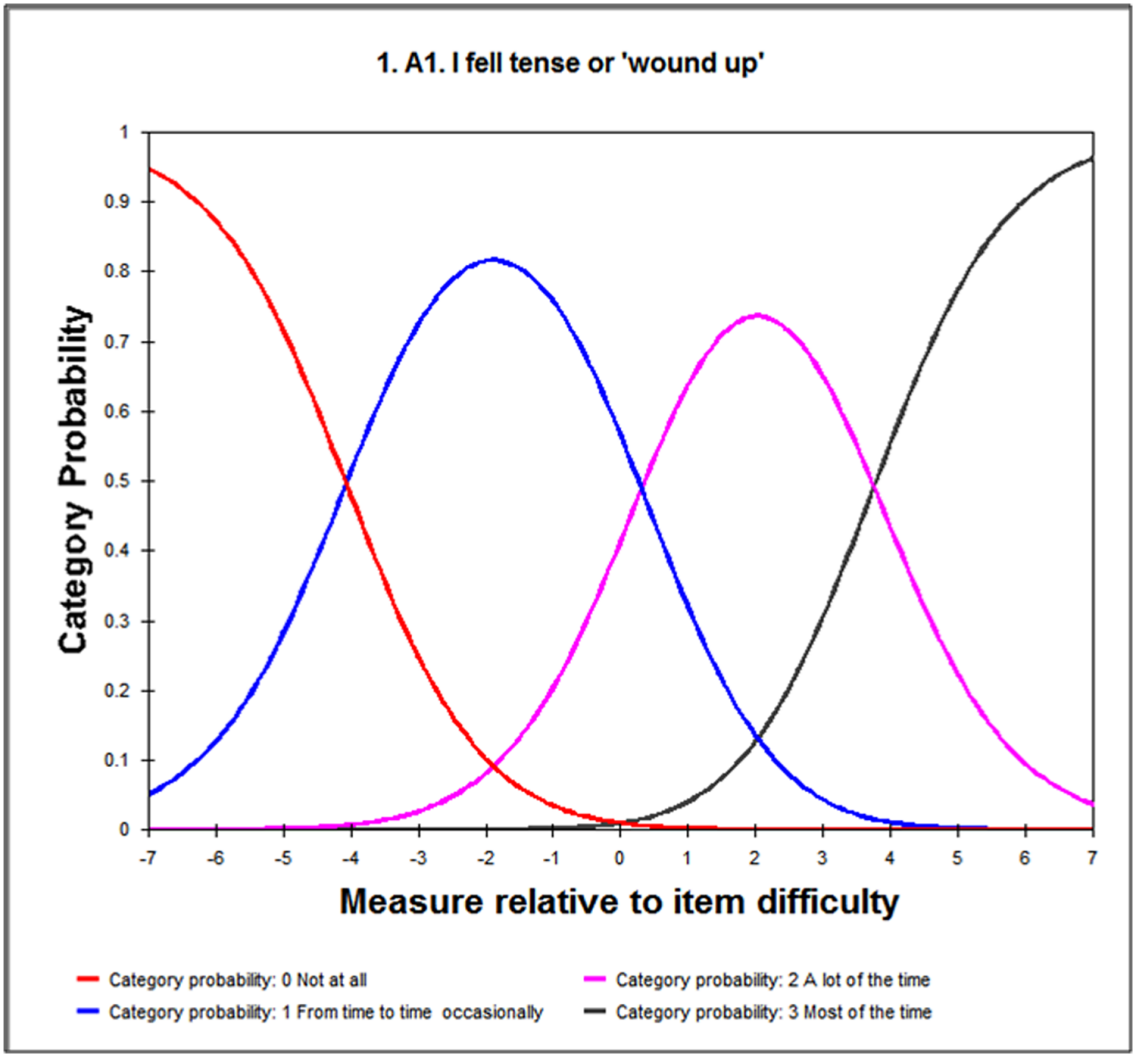
Example of the category probability curves: HADS-A1.

**Fig 2:**
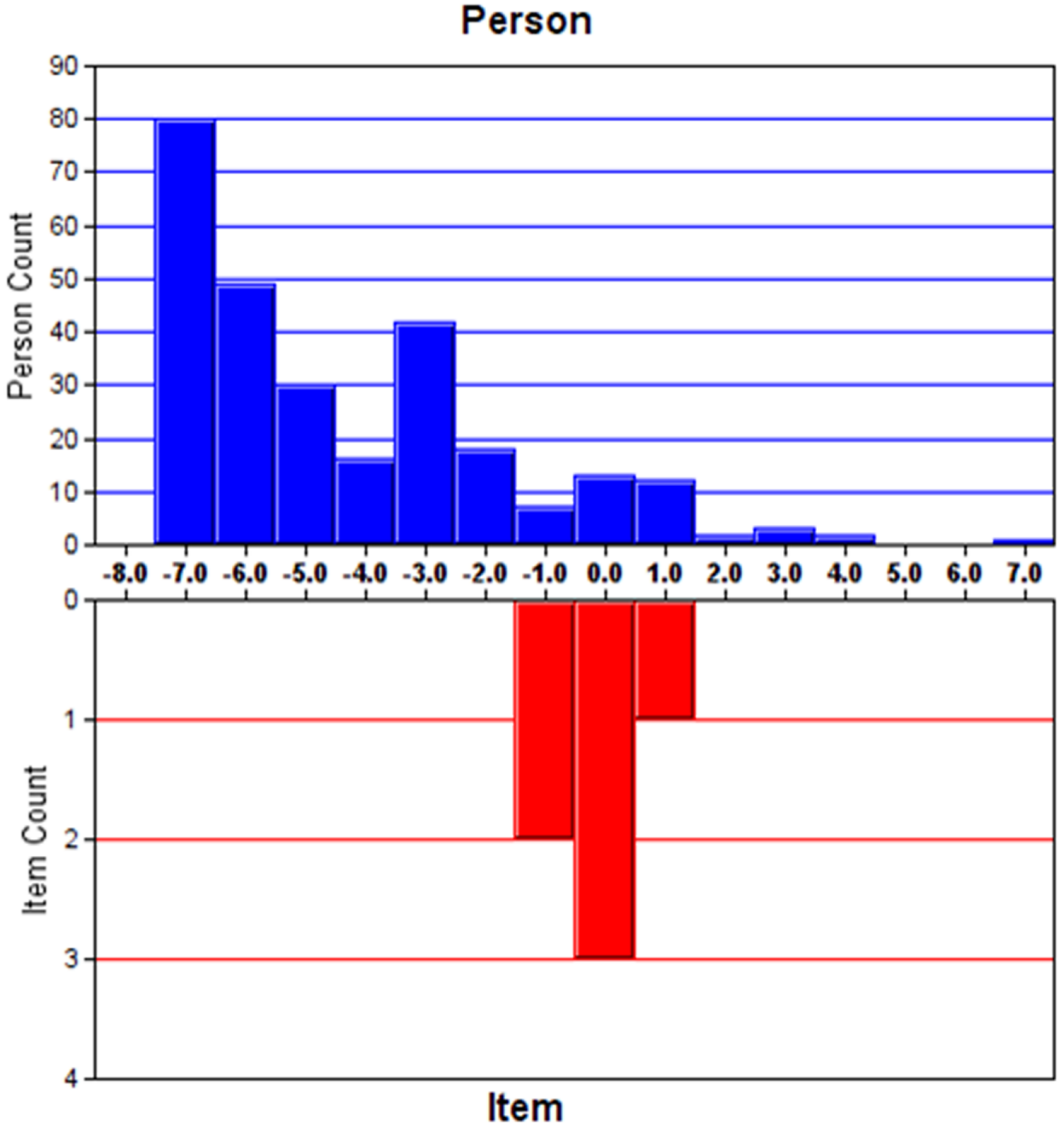
Person-item location distribution for HADS-Anxiety.

Analysis of the initial HADS- Anxiety (HADS-A) items shows the mean infit MNSQ value for items A7 (I can sit at ease and feel relaxed) was 1.44, indicating misfit the Rasch model(infit MNSQ = 1.44 and outfit MNSQ = 1.37), outside an ideal MNSQ value ranges between 0.7 and 1.3. Removal of item A7 significantly improved the model fit (chi square of χ^2^(10) = 452.4, P<0.001) when comparing with the initial model by likelihood ratio test (Table 2). The modified HADS-A exhibited a PSI of 1.95 and PSR of 0.79, suggesting a good discriminant ability of the questionnaire. The instrument appeared to be on target even though 1.22 error of person measure was slightly higher than the suggested value. The residuals explained 61.0% of the raw variance and 5.57% of the significant t-tests indicated unidimensionality. The unexplained variance in 1st contrast was 1.57 eigenvalue units, showing no evidence of another latent trait captured by the scale. The category probability curves for HADS-A revealed that all items of subscales had ordered thresholds (Fig 1). But the skewed distribution of person ability may affect fit statistic (Fig 2), which was expected to be normally distributed. No local dependency was detected with all paired standardized residual correlations <0.30.

**Table 2:** Summary statistics for Rasch analysis of the initial and revised structures.

Action	# of items	Rasch model fit			Targeting (SE of person measure)	Measurement precision		Local dependency	Unidimensionality		
		Infit MNSQ Mean (Range)	Outfit MNSQ Mean (Range)	Liklihood ratio test¶		PSR	PSI		%Significant t-test	% Variance explained by measures	Unexplained variance in 1st contrast (Eigenvalue)
**HADS-Anxiety**											
Initial	7	1.00 (0.68–1.44)	0.98 (0.73–1.37)	/	1.10	0.78	1.89	No	5.05%	58.7	1.67
Remove item A7*	6	1.01 (0.73–1.29)	1.00 (0.73–1.32)	χ^2^ (10) = 452.4 P<0.001	1.22	0.79	1.95	No	5.57%	61.0	1.57
**HADS-Depression**											
Initial	7	0.99 (0.74–1.34)	0.99 (0.74–1.25)	/	0.88	0.77	1.82	No	4.97%	60.9	1.54
Item D14 rescored (0112)	7	0.99 (0.73–1.22)	0.99 (0.57–1.29)	χ^2^ (2) = 212.0 P<0.001	0.93	0.78	1.88	No	5.03%	61.9	1.55
**HADS-Total**											
Initial†	13	0.99 (0.73–1.33)	0.94 (0.59–1.33)	/	0.68	0.86	2.52	No	4.88%	59.5	2.10
Remove item D12‡	12	0.99 (0.71–1.29)	0.94 (0.58–1.30)	χ^2^ (5) = 289.3 P<0.001	0.71	0.85	2.37	No	4.79%	59.4	2.05
**Ideal values**		***1*.*00 (0*.*70–1*.*30)***	***1*.*00 (0*.*70–1*.*30)***	***P<0*.*05***	***<1 error***	***≥0*.*80***	***≥2*.*00***	**No positive correlation** **>0.30**	***<5*.*00%***	***>60*.*0***	***<2*.*00***

Infit MNSQ = Infit Mean-Square; Outfit MNSQ = Outfit Mean-Square; SE = Standard Error; PSR = Person Separation Reliability; PSI = Person Separation Index

*The item A7 was removed with infit MNSQ = 1.44 indicating misfit the Rasch model.

^†^Initial fit using 13 items from the modified HADS-Anxiety and HADS-Depression subscales. Item D14 was rescored in the same manner as the HADS-Depression analysis.

^‡^Item D12failed to load on depression subscales but instead loaded with the anxiety items. Item D12 was removed.

^¶^The current model was compared with the model in the last action.

Table 3 shows the individual item fit statistics and scoring structure for the modified HADS-A. All items of HADS-A were free from uniform DIF (S1 Table). Notable non-uniform DIF was detected on items A9(“Get a sort of frightened feeling like ‘butterflies’ in the stomach”) and A11(“Feel restless as I have to be on the move”) by education subgroups in high ability group with DIF difference of 1.14 and -1.20 respectively which indicated that item A9 was more difficult for people with education of primary school or lower than those with higher education in high ability group, while item A11 was more difficult for people with higher education in high ability group (S2 Table).

**Table 3:** Item fit statistics and scoring structure for HADS-Anxiety.

Item	Description	Measure	SE	Infit MNSQ	Loading	Scoring structure
HADS-A1	Tense	-1.25	0.15	1.02	0.75	3-2-1-0
HADS-A3	Frightened feeling	-0.73	0.15	0.99	0.56	3-2-1-0
HADS-A5	Worrying thoughts	1.12	0.17	1.29	-0.46	3-2-1-0
HADS-A9	Butterflies	0.42	0.16	1.05	-0.64	0-1-2-3
HADS-A11	Restless	0.15	0.16	0.95	-0.16	3-2-1-0
HADS-A13	Panic	0.28	0.16	0.73	-0.22	3-2-1-0

SE = Standard Error; Infit MNSQ = Infit Mean-Square.

### Rasch analysis of HADS-Depression

The respondents may have difficulty to discriminate two response categories between “Sometimes” and “Not often” on the item D14 (Enjoy book or radio or TV). Thus, we rescored items D14 by adjoining the 2^nd^ and 3^rd^ categories (the new scoring structure 0-1-1-2, Table 4). The modified model provided a better fit to the data than the original model (chi square of χ^2^(2) = 212.0, *P*<0.001) (Table 2). A PSI of 1.88 and a PSR of 0.78 suggested good discriminant ability of the questionnaire. The instrument appeared to be on good target with 0.93 error of person measure. The residual explained 61.9% of the raw variance and 5.03% significant t-tests indicated that no multidimensionality appeared in the scale. The unexplained variance in 1st contrast was 1.55 eigenvalue units. All items of subscales had ordered thresholds (Figure was not shown).

**Table 4:** Item fit statistics and scoring structure for HADS-Depression.

Item	Description	Measure	SE	Infit MNSQ	Loading	Scoring structure
HADS-D2	Enjoy things	0.34	0.12	1.19	0.42	0-1-2-3
HADS-D4	Laugh	-0.27	0.11	0.77	-0.20	0-1-2-3
HADS-D6	Cheerful	0.22	0.12	0.73	-0.60	3-2-1-0
HADS-D8	Slowed down	-0.07	0.12	1.22	0.67	3-2-1-0
HADS-D10	Interest in appearance	0.49	0.13	0.85	-0.51	3-2-1-0
HADS-D12	Enjoyment	1.39	0.14	0.98	-0.42	0-1-2-3
HADS-D14	Enjoy book or radio or TV	-2.10	0.13	1.22	0.29	0-1-1-2

SE = Standard Error; Infit MNSQ = Infit Mean-Square.

**Fig 3:**
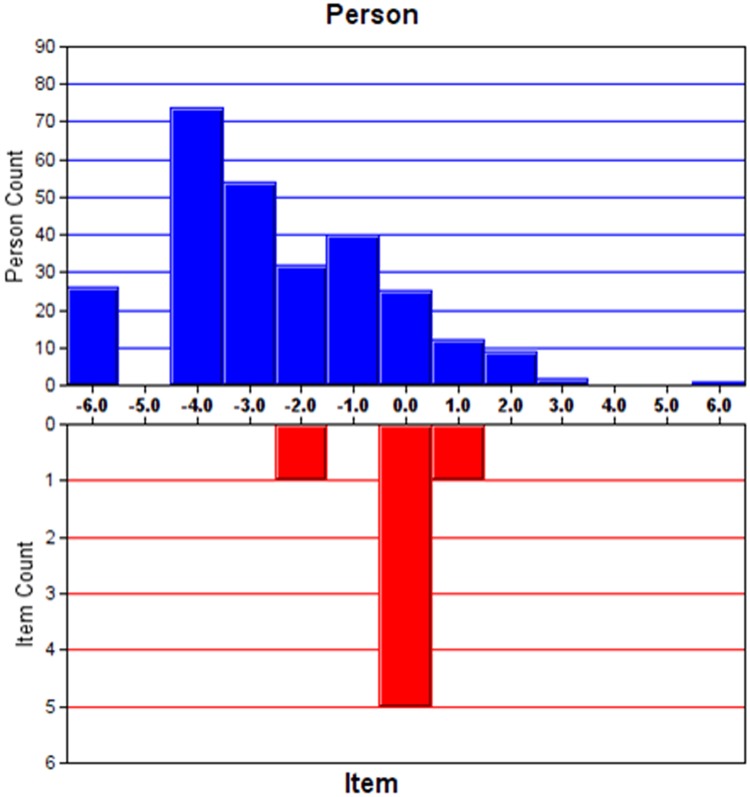
Person-item location distribution for HADS- Depression.

Table 4 shows the individual item fit statistics and scoring structure for the modified HADS-D. Fig 3 shows a slightly skewed Person-item location distribution. No local dependency was detected with all paired standardized residual correlations <0.30. All items of HADS-D were free from both uniform and non-uniform DIF (S1 and S2 Tables).

### Rasch analysis of HADS-Total

**Fig 4:**
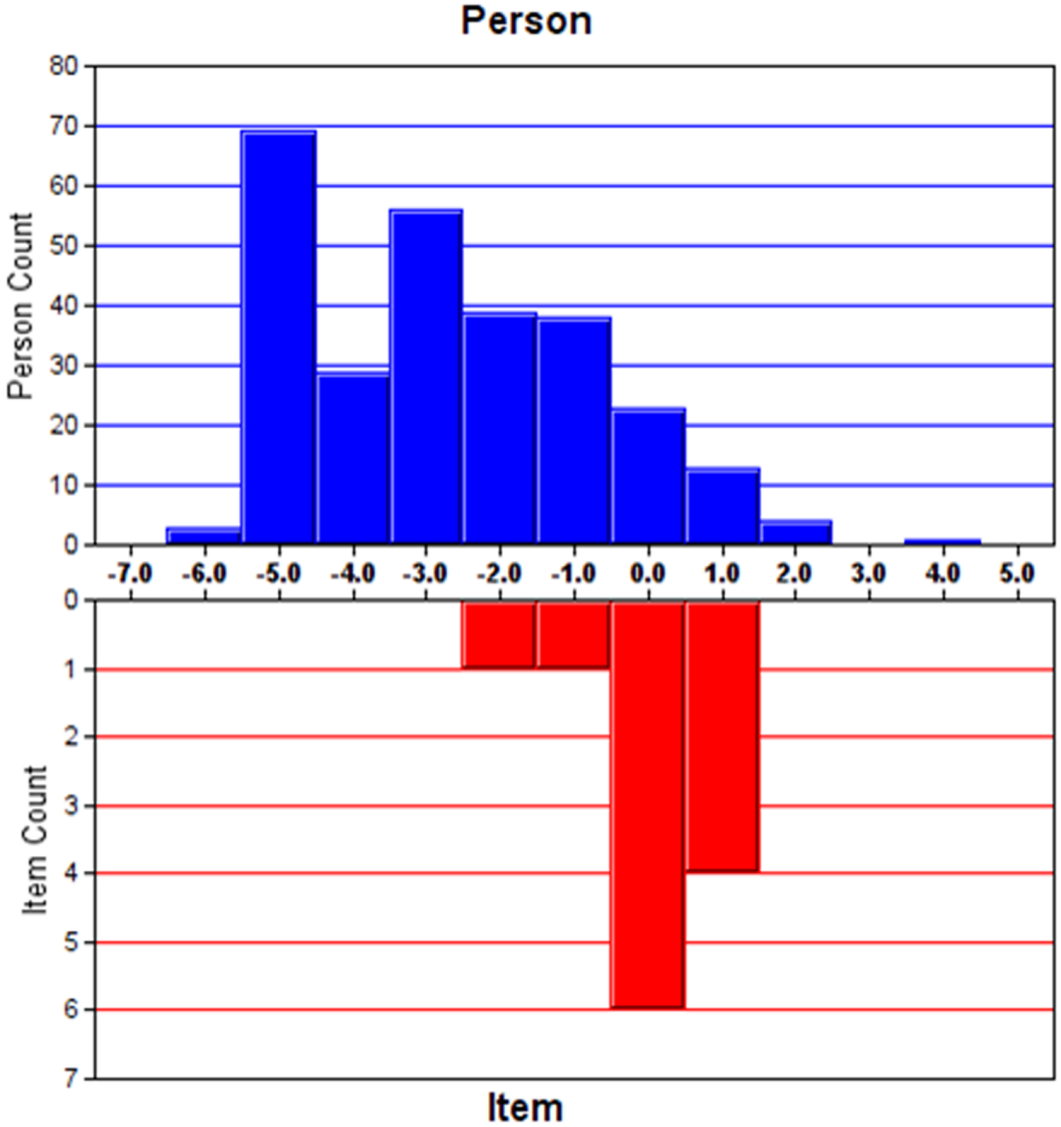
Person-item location distribution for HADS- Total.

The analysis of Initial HADS-Total (HADS-T) fit started with 13 items from the modified HADS-A and HADS-D subscales. Item D14 was rescored in the same way as it was in HADS-D analysis. Item D12 (I look forward with enjoyment to things) failed to load on the depression subscales but instead loaded with the anxiety items. Thus, item D12 was removed. This resulted in an improved fit to the Rasch model (χ^2^((5) = 289.3, P<0.001, Table 2). A PSI of 2.37 and a PSR of 0.85 suggested good discriminant ability of the questionnaire. The instrument appeared to be on good target with 0.71 error of person measure. The residual explained 59.4% of the raw variance 4.79% significant t-tests indicated unidimentionality. The unexplained variance in 1st contrast was 2.05 eigenvalue units. No local dependency was detected with all paired standardized residual correlations <0.30. Table 5 shows the individual item fit statistics and scoring structure for the modified HADS-T version. Fig 4 shows a slightly skewed Person-item location distribution for HADS-T.

**Table 5:** Item fit statistics and scoring structure for HADS-Total.

Item	Description	Measure	SE	Infit MNSQ	Loading	Scoring structure
HADS-A1	Tense	-0.07	0.12	1.16	0.57	3-2-1-0
HADS-D2	Enjoy things	-0.09	0.12	1.22	-0.37	0-1-2-3
HADS-A3	Frightened feeling	0.06	0.12	1.07	0.58	3-2-1-0
HADS-D4	Laugh	-0.59	0.11	0.96	-0.45	0-1-2-3
HADS-A5	Worrying thoughts	0.80	0.14	0.92	0.22	3-2-1-0
HADS-D6	Cheerful	-0.17	0.12	0.71	-0.16	3-2-1-0
HADS-D8	Slowed down	-0.34	0.12	1.28	-0.51	3-2-1-0
HADS-A9	Butterflies	0.71	0.13	0.79	0.37	0-1-2-3
HADS-D10	Interest in appearance	0.11	0.12	0.97	-0.13	3-2-1-0
HADS-A11	Panic feeling	0.79	0.14	0.82	0.43	3-2-1-0
HADS-A13	Panic	1.11	0.14	0.74	-0.41	3-2-1-0
HADS-D14	Enjoy book or radio or TV	-2.32	0.12	1.29	-0.45	0-1-1-2

SE = Standard Error; Infit MNSQ = Infit Mean-Square.

All items of HADS-T were free from uniform DIF (S1 Table). Notable non-uniform DIF was detected on items A1 (“Feel tense or ‘wound up’”) and D14 (“Enjoy book or radio or TV”) by sex and education subgroups in high ability group. Item A1 was more difficult for female (DIF contrast = -1.17) and people with higher education (DIF contrast = -1.10) in high ability group. Item D14 was more difficult for male (DIF contrast = 1.12) and people with higher education (DIF contrast = -1.64) in high ability group (S2 Table).

### Modified cut-off points

**Fig 5:**
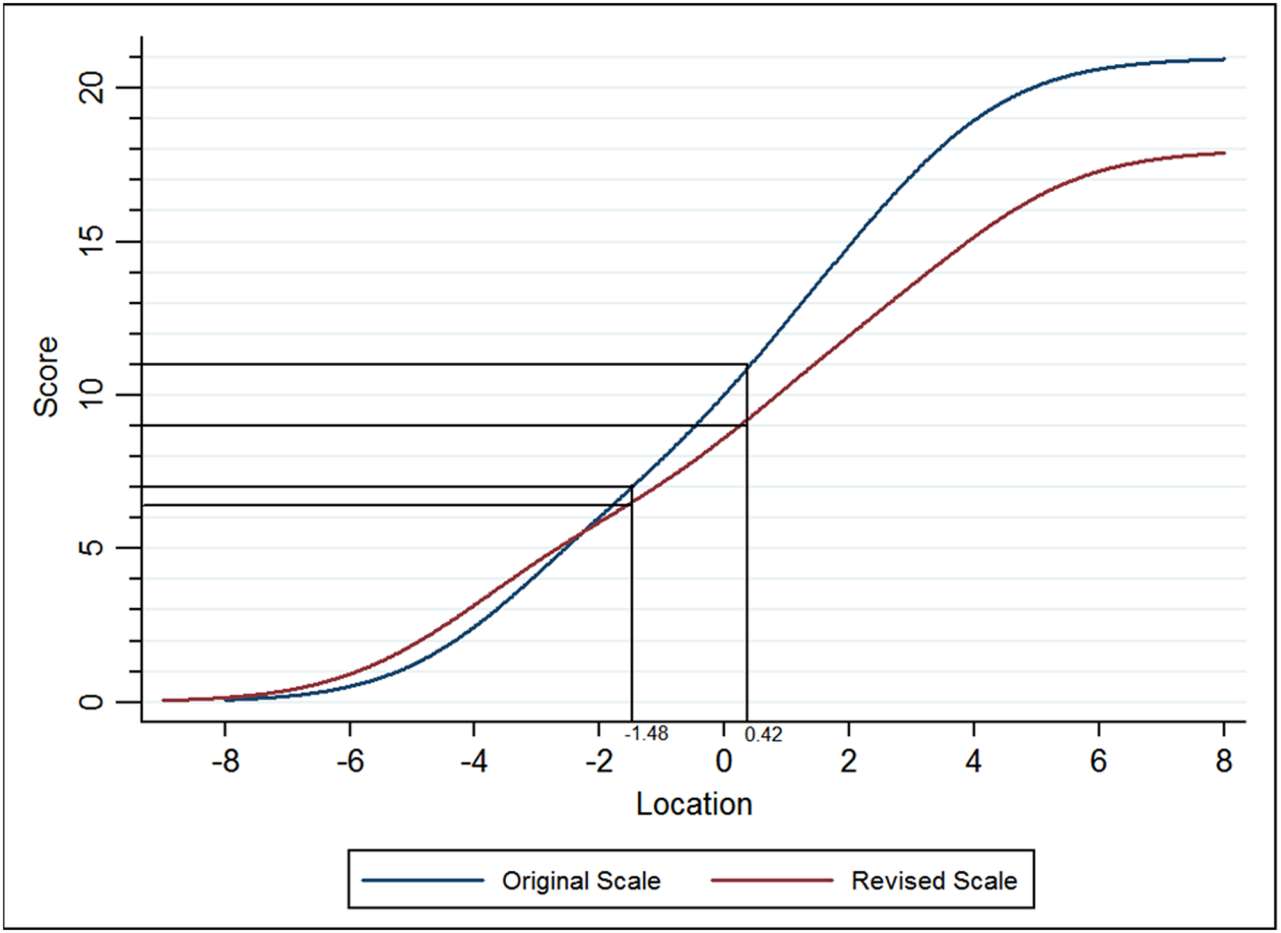
Equating tests to ascertain new cut-off scores for the HADS-A.

**Fig 6:**
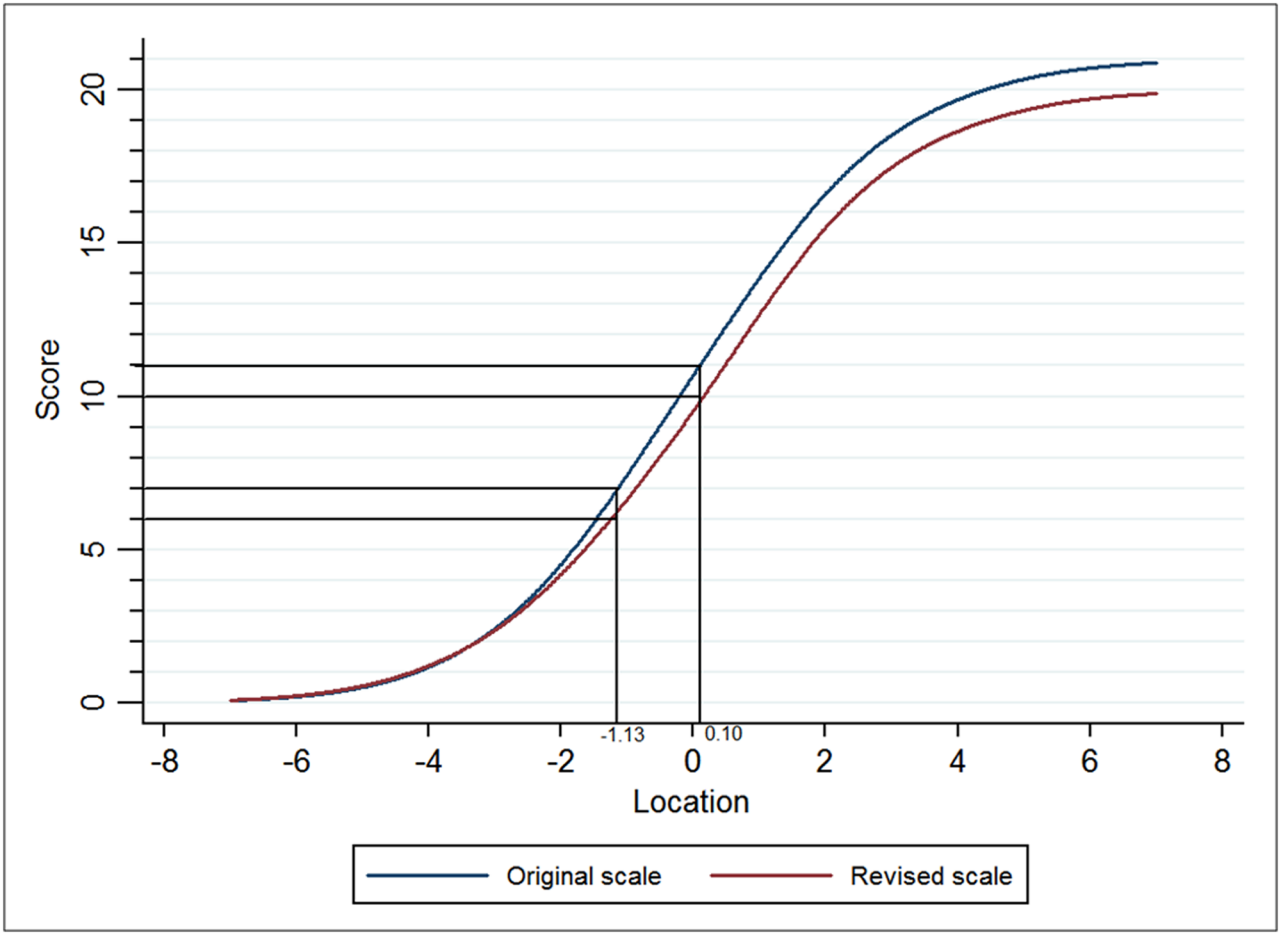
Equating tests to ascertain new cut-off scores for the HADS-D.

Table 6 shows the relationship between cut-off points on the original scale and on the revised scale. Equating tests gave new upper and lower cut-off points of 9 and 6 for the modified HADS-A, while 10 and 6 for HADS-D (Figs 5 and 6).

**Table 6:** Equated cut-off points.

	Original cut-off	N	%	Revised cut-off	N	%
HADS-A*	≥11	19	7.22	≥9	21	7.98
	8–10	17	6.46	7–8	10	3.80
	≤7	227	86.3	≤6	232	88.2
**HADS-D**†	≥11	27	10.1	≥10	30	11.2
	8–10	36	13.5	7–9	36	13.5
	≤7	204	76.4	≤6	201	75.3

* 12 subjects had missing HADS-A data.

^†^8 subjects had missing HADS-D data.

Table 6 shows that the original HADS-A overestimated the prevalence of borderline abnormal anxiety. The equating scores for HADS-D subscale showed that rescoring D14 had no effect on the prevalence of all three levels of depression.

## Discussion

The depression and/or anxiety among cataract patients in different countries have been assessed using diverse screening instruments, including HADS in the study of Japan[32], PHQ-9 in China[8], GADS[9] and the Center for Epidemiologic Studies Depression Scale (CESD)[12] in Australia and the Geriatric Depression Scale (GDS) in Canada[33]. The HADS have been also used in patients with other ocular diseases such as glaucoma, AMD, dry eye and ptosis[34]. All the instruments are self-administered, and the contents are close to everyday activities and speech, but the HADS is shorter than the GADS, CESD and GDS, and it evaluates anxiety and depression on two separated parts. Our results demonstrate that the HADS is a unidimensional, reliable and valid instrument for assessing anxiety and depression in Chinese cataract population. As indicated by Rasch analysis, the standard 7-item measure of anxiety and depression subscales should be modified for use in cataract patients. The modified HADS subscales had ordered thresholds and there was no evidence of large DIF.

Our results found item A7 (I can sit at ease and feel relaxed) misfit the Rasch model, and removal of item A7 improved model and provided a better fit for HADS-A, as previous studies suggested[17, 19]. Previous studies have found Item A7 either loaded on HADS-D subscale or was complex, with some analysis showing higher loading on HADS-D subscale[19, 35]. It might be that item A7 included the positive wording, which was corresponding with the positively worded items of HADS-D subscale[19, 36]. For HADS-D, we rescored the item D14 (“Enjoy a good book or radio or TV program”) (0-1-1-2) to improve the model fit. Traditional factor analysis have found Item D12 (I look forward with enjoyment to things) to load highly on the factor corresponding to HADS-D subscale[19]. However, in our study, D12 was found to load strongly with Anxiety items, and removal of item D12 may be reasonable from a clinical perspective. The level of reliability of these modified subscales makes it suitable for estimation of anxiety and depression states in Chinese cataract patients.

Therefore, we ascertained the new cut-off points for the modified version of HADS. The cut-off points may be useful for clinicians to make clinical decision. Our current result showed that the original cut-off points of anxiety and depression subscales misestimated anxiety and depression states for cataract patients in China. Using the revised cut-off points, the ratios of anxiety and depression in cataract patients were increasing.

The current study demonstrated no uniform DIF and no non-uniform DIF for the majority of the items, indicating the measures were not affected by item bias (age, sex, education). However, items A9 (“Get a sort of frightened feeling like ‘butterflies’ in the stomach”), A11 (“Feel restless as I have to be on the move”) for HADS-A and A1 (“Feel tense or ‘wound up’”), D14 (“Enjoy book or radio or TV”) for HADS-T had a notable non-uniform DIF between lower and higher education subgroups at high ability level. It is possible that people with lower education are more likely to experience nervous tension or difficulty in reading activity. In addition, item A1 was more likely to be endorsed by male. A study showed the reduction in vision-related emotional well-being was significantly greater in men compared with women[37]. And item D14 was more likely to be endorsed by female. It is possible that these activities are more commonly performed among females in China.

There were some limitations in this study. First, the study sample was recruited from a hospital in Southern China and is not completely representative of its general population in China. Second, although the HADS is easy and convenient for study purposes, it is not comparable with a formal psychiatric diagnosis of depression or anxiety.

In conclusion, the modified version of HADS has been shown to be a reliable and valid instrument, and is useful for assessing anxiety and depression in Chinese cataract population.

## Supporting information

S1 Table Uniform differential item functioning (DIF) assessed by age, sex, education. (DIF difference>1.0 logits was in bold to indicate that uniform DIF would occur)*.
(PDF)

S2 Table Non-uniform differential item functioning (NUDIF) assessed by age, sex, education. (NUDIF difference>1.0 logits was in bold to indicate that non-uniform DIF would occur)*.
(PDF)
