# Supplementary material for: Rasch analysis of the hospital anxiety and depression scale among Chinese cataract patients
Source: PLoS One. 2017 Sep 26;12(9):e0185287. doi: 10.1371/journal.pone.0185287 (PMC5614566; doi:10.1371/journal.pone.0185287)
Supplement: S1 Table — (DIF difference>1.0 logits was in bold to indicate that uniform DIF would occur)*. (PDF) [file pone.0185287.s001.pdf]

S1 Table. Uniform differential item functioning (DIF) assessed by age, sex, education.(DIF difference>1.0 logits was in bold to indicate that uniform DIF would occur.)*

	HADS-Anxiety						HADS-Depression						
	**A1**	**A3**	**A5**	**A9**	**A11**	**A13**	**D2**	**D4**	**D6**	**D8**	**D10**	**D12**	**D14**
**Age (≤70 - >70)**	-0.06	-0.07	0.28	0.16	-0.26	0.03	-0.33	0.00	-0.56	0.84	0.63	-0.15	-0.49
**Sex (Male - Female)**	0.00	-0.05	-0.19	-0.12	0.10	0.23	0.52	0.09	-0.69	0.26	-0.19	-0.49	0.34
**Education(≤Primary school - ≥Junior school)**	-0.38	-0.41	0.69	0.21	-0.31	0.42	-0.23	0.26	0.24	-0.39	0.34	-0.21	-0.05

	HADS-Total											
	**A1**	**A3**	**A5**	**A9**	**A11**	**A13**	**D2**	**D4**	**D6**	**D8**	**D10**	**D14**
**Age (≤70 - >70)**	-0.20	-0.03	0.00	-0.17	-0.11	-0.12	-0.21	0.06	-0.34	0.66	0.64	-0.31
**Sex (Male - Female)**	-0.24	-0.21	-0.31	-0.42	0.00	-0.08	0.62	0.18	-0.41	0.26	-0.08	0.37
**Education(≤Primary school - ≥Junior school)**	-0.35	-0.45	0.40	0.24	-0.34	0.25	-0.20	0.29	0.15	-0.10	0.34	-0.12

*A positive DIF contrast indicates that the item is more difficult for the left-hand-listed CLASS.
