# Supplementary material for: Rasch analysis of the hospital anxiety and depression scale among Chinese cataract patients
Source: PLoS One. 2017 Sep 26;12(9):e0185287. doi: 10.1371/journal.pone.0185287 (PMC5614566; doi:10.1371/journal.pone.0185287)
Supplement: S2 Table — (NUDIF difference>1.0 logits was in bold to indicate that non-uniform DIF would occur)*. (PDF) [file pone.0185287.s002.pdf]

S2 Table. Non-uniform Differential item functioning (NUDIF) assessed by age, sex, education. (NUDIF difference>1.0 logits was in bold to indicate that non-uniform DIF would occur.)*

	HADS-Anxiety						HADS-Depression						
	**A1**	**A3**	**A5**	**A9**	**A11**	**A13**	**D2**	**D4**	**D6**	**D8**	**D10**	**D12**	**D14**
**Age (≤70 - >70)**													
Low ability	-0.24	-0.11	0.45	0.40	-0.28	-0.02	-0.34	-0.15	-0.51	0.89	0.86	-0.17	-0.48
High ability	0.42	-0.05	0.43	-0.33	-0.36	-0.06	-0.28	0.18	-0.76	0.67	0.34	-0.11	-0.54
**Sex (Male - Female)**													
Low ability	0.22	-0.18	-0.25	-0.13	0.11	0.07	0.50	0.20	-1.00	0.26	-0.12	-0.64	0.36
High ability	-1.00	0.45	-0.07	0.02	-0.04	0.72	0.45	-0.11	-0.09	0.11	-0.26	-0.04	-0.01
**Education (≤Primary school - ≥Junior school)**													
Low ability	-0.18	-0.48	0.44	-0.21	-0.01	0.60	-0.30	0.30	0.10	-0.21	0.29	-0.04	-0.06
High ability	-1.00	0.05	0.61	**1.14**	**-1.20**	0.25	0.10	0.14	0.58	-0.58	0.38	-0.92	0.01

	HADS-Total											
	**A1**	**A3**	**A5**	**A9**	**A11**	**A13**	**D2**	**D4**	**D6**	**D8**	**D10**	**D14**
**Age (≤70 - >70)**												
Low ability	-0.05	0.02	0.00	0.00	-0.01	-0.01	0.00	-0.01	-0.07	0.11	0.04	-0.03
High ability	0.11	-0.21	0.00	-0.14	-0.04	0.00	-0.24	0.14	0.18	-0.09	0.32	-0.03
**Sex (Male - Female)**												
Low ability	0.04	-0.28	-0.11	-0.38	0.07	0.09	0.53	0.09	-0.71	0.24	-0.36	0.28
High ability	**-1.17**	-0.02	-0.58	-0.52	-0.22	-0.64	0.76	0.41	0.33	0.18	0.43	**1.12**
**Education (≤Primary school - ≥Junior school)**												
Low ability	-0.24	-0.40	0.50	0.00	-0.28	0.24	-0.19	0.25	0.12	-0.04	0.19	0.05
High ability	**-1.10**	-0.50	0.09	0.76	-0.86	0.16	0.06	0.57	0.06	0.25	0.65	**-1.64**

*A positive DIF contrast indicates that the item is more difficult for the left-hand-listed CLASS.
